# Insulin as a Primary Autoantigen for Type 1A Diabetes

**DOI:** 10.1080/17402520500078204

**Published:** 2005-09

**Authors:** J. M. Jasinski, G. S. Eisenbarth

**Affiliations:** 1 Human Medical Genetics Program 1775 North Ursula Street Aurora CO 80010 USA; 2 Barbara Davis Center for Childhood Diabetes 1775 North Ursula Street Aurora CO 80010 USA; 3 University of Colorado Health Sciences Center 1775 North Ursula Street Aurora CO 80010 USA

## Abstract

Type 1A diabetes mellitus is caused by specific and progressive autoimmune destruction of the beta cells in the islets of Langerhans whereas the other cell types in the islet (alpha, delta, and PP) are spared. The autoantigens of Type 1A diabetes may be divided into subgroups based on their tissue distributions: Beta-cell-specific antigens like insulin, insulin derivatives, and IGRP (Islet-specific Glucose-6-phosphatase catalytic subunit Related Peptide); neurendocrine antigens such as carboxypeptidase H, insulinoma-associated antigen (IA-2), glutamic acid decarboxylase (GAD65), and carboxypeptidase E; and those expressed ubiquitously like heat shock protein 60 (a putative autoantigen for type 1 diabetes). This review will focus specifically on insulin as a primary autoantigen, an essentia l target for disease, in type 1A diabetes mellitus. In particular, immunization with insulin peptide B:9-23 can be used to induce insulin autoantibodies and diabetes in animal models or used to prevent diabetes. Genetic manipulation of the insulin 1 and 2 genes reciprocally alters development of diabetes in the NOD mouse, and insulin gene polymorphisms are important determinants of childhood diabetes. We are pursuing the hypothesis that insulin is a primary autoantigen for type 1 diabetes, and thus the pathogenesis of the disease relates to specific recognition of one or more peptides.


*Clinical & Developmental Immunology*, September 2005; 12(3): 181–186

Taylor & Francis

Taylor & Francis Group

### Insulin as a primary autoantigen for type 1A diabetes

J. M. JASINSKI[1,2,†], & G. S. EISENBARTH[2,3]

[1]*Human Medical Genetics Program, 1775 North Ursula Street, Aurora, CO 80010, USA,* [2]*Barbara Davis Center for Childhood Diabetes, 1775 North Ursula Street, Aurora, CO 80010, USA, and* [3]*University of Colorado Health Sciences Center, 1775 North Ursula Street, Aurora, CO 80010, USA*

**Abstract**
Type 1A diabetes mellitus is caused by specific and progressive autoimmune destruction of the beta cells in the islets of Langerhans whereas the other cell types in the islet (alpha, delta, and PP) are spared. The autoantigens of Type 1A diabetes may be divided into subgroups based on their tissue distributions: Beta-cell-specific antigens like insulin, insulin derivatives, and IGRP (Islet-specific Glucose-6-phosphatase catalytic subunit Related Peptide); neurendocrine antigens such as carboxypeptidase H, insulinoma-associated antigen (IA-2), glutamic acid decarboxylase (GAD65), and carboxypeptidase E; and those expressed ubiquitously like heat shock protein 60 (a putative autoantigen for type 1 diabetes). This review will focus specifically on insulin as a primary autoantigen, an essential target for disease, in type 1A diabetes mellitus. In particular, immunization with insulin peptide B:9-23 can be used to induce insulin autoantibodies and diabetes in animal models or used to prevent diabetes. Genetic manipulation of the insulin 1 and 2 genes reciprocally alters development of diabetes in the NOD mouse, and insulin gene polymorphisms are important determinants of childhood diabetes. We are pursuing the hypothesis that insulin is a primary autoantigen for type 1 diabetes, and thus the pathogenesis of the disease relates to specific recognition of one or more peptides.

**Keywords:** *Autoantigen, autoimmune, insulin, type 1 diabetes*

### Introduction

Type 1 diabetes mellitus (T1DM) is characterized by islet beta cell destruction and the loss of insulin secretion. The etiology of type 1A diabetes mellitus is autoimmune, and autoantibodies may be detected years before overt disease is diagnosed (Juhl and Hutton). By contrast, type 1B diabetes mellitus is defined as diabetes resulting from the loss of insulin secretion but is not immune-mediated. Type 1A diabetes is a complex disorder involving genetic and environmental interactions. Type 1A diabetes fulfills the classical criteria for autoimmune disease (Milgrom and Witebsky 1962). Humoral autoimmunity develops over a period of months to years (Yu et al. 1996) following a mostly hypothetical precipitating environmental insult (Sairenji et al. 1991, Graves et al. 2003, Norris et al. 2003, Stene et al. 2004) in genetically susceptible individuals. The proposed model for diabetes development in Figure 1 suggests that an individual may experience several years of undetected beta cell loss before a functional deficiency of insulin secretion results in hyperglycemia. Recently, the BABYDIAB project published findings that autoantibodies can be detected as early as nine months of age in offspring of diabetic parents (Hummel et al. 2004). Specific autoantigens such as insulin, proinsulin, insulin peptide B:9-23, GAD65, IA-2, islet gangliosides (GM2-1), phogrin (IA-2 beta), IGRP, and IA-2 have been identified (Yu and Eisenbarth, Wegmann et al. 1994, Dotta et al. 1996, Wong et al. 1999, Hutton and Eisenbarth 2003). Insulitis preceding disease has been demonstrated repeatedly in multiple animal models (Yu and Eisenbarth). Multiple experiments have shown the diabetogenicity of splenic CD4 and CD8 T cells transferred to animals with and without an autoimmune background or to *scid* mice

Correspondence: G. S. Eisenbarth, Barbara Davis Center for Childhood Diabetes, University of Colorado Health Sciences Center, Mail Stop B140, PO Box 6511, Aurora, CO 80045-6511, USA. Tel: 1 303 724 6847. Fax: 1 303 724 6830. E-mail: george.eisenbarth@uchsc.edu
†Tel: 1 303 724 6812. Fax: 1 303 724 6847. E-mail: jean.jasinski@uchsc.edu

ISSN 1740-2522 print/ISSN 1740-2530 online © 2005 Taylor & Francis
DOI: 10.1080/17402520500078204

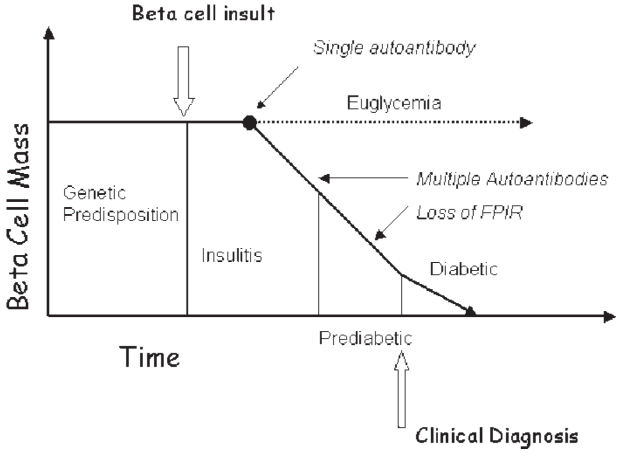

Figure 1. Chronic model for development of type 1 diabetes. A genetically susceptible individual experiences a triggering event resulting in insulitis. Due to antigen shedding or inflammation, insulitis may be followed by development of autoantibodies. Some individuals (likely those with single antibody positivity) do not progress to diabetes (Hummel et al. 2004) (dotted line) whereas others experience a slow loss of beta cell mass and insulin secretion, leading to the development of overt disease. FPIR (first phase insulin response) is a measure of residual beta cell function. Figure adapted from Teaching Slides (Yu and Eisenbarth) and Eisenbarth et al. (1988). The kinetics of the development of diabetes may be different in different populations (von Herrath and Bach 2002).

(Wegmann et al. 1994, Wong et al. 1999, Thebault-Baumont et al. 2003).

Unlike other human autoimmune diseases like Graves Disease or myasthenia gravis, autoantibodies are not by themselves pathogenic (Marner et al. 1985, Martin et al. 2001). Rather they serve as a surrogate marker of beta cell damage and insulitis (Eskola et al. 2003), although studies in the NOD mouse indicate that transplacental antibodies greatly enhance progression to diabetes (Greeley et al. 2002).

The number of different biochemical autoantibodies (GAD65, IA-2, insulin) is a strong predictor of future disease development (Verge et al. 1998, Hummel et al. 2004). Those individuals expressing 2 or more autoantibodies have a 39% risk of developing diabetes within 3 years and a 68% risk of disease within 5 years. In a small study, all first-degree relatives expressing all three autoantibodies developed diabetes within 5 years (Verge et al. 1996). Detected via staining of patient serum and frozen human pancreas, cytoplasmic islet cell antibodies (ICA) in unaffected first-degree relatives of diabetics confer a 30–50% risk of developing clinical diabetes within 5–10 years (Eskola et al. 2003). The earlier the autoantibodies appear in children of type 1 diabetic parents, the more likely and quickly a child is to progress to multiple autoantibody positivity and diabetes. The different autoantibody types generally appear sequentially rather than simultaneously (Yu et al. 1996) with insulin generally appearing first in young persons developing Type 1A diabetes. Approximately 10–15% of all adults with diabetes may have LADA (latent autoimmune diabetes of adults) as evidenced by the presence of GAD autoantibodies (Zimmet et al. 1999).

### Effector cells in type 1A diabetes

In order to develop a T-cell mediated autoimmune disease such as type 1 diabetes, at least three essential immune cell types are necessary. Antigen presenting cells (APCs) must be capable of presenting self-antigens via MHC (major histocompatibility complex) molecules. There must be T cells that recognize self-peptides. Lastly in diseases where autoantibodies are present, there must be B cells that produce autoantibodies usually to intact self-proteins.

### *Antigen presentation*

APCs only present peptides bound to MHC molecules. Approximately 40–50% of the genetic risk for diabetes is attributed to alleles of the MHC genes (Wucherpfennig 2003). The peptide-binding groove of an MHC molecule directly influences which peptides can be presented by a specific MHC allele. As the crystal structure of DQ8 and insulin peptide shows, some high-risk Class II MHC alleles (DR3/4 and DQ2/8 in humans and IA$^{g7}$ in mice) bind and present insulin peptides with interesting and distinct properties. NOD and some high-risk human alleles do not have a negatively charged amino acid in position 57 of the MHC beta chain unlike other MHC types that have an aspartic acid in this position (Wucherpfennig 2003). This creates a net positive charge in the P9 binding pocket of the peptide-binding groove that accommodates peptides from the B chain of insulin. Class I MHC of the NOD mice (K$^{d}$) can bind residues 15-23 of the B chain of insulin (B:15-23) and I-A$^{g7}$ (class II) can bind B:9-23. In fact, the P1 and P9 pockets of I-A$^{g7}$ bind to anchor residues Glu13 and Glu21 in insulin whereas other MHC molecules favor hydrophobic side chains in these positions. Previous experiments that concluded that I-A$^{g7}$ bound peptides poorly were based on I-A$^{g7}$ dissociating from its cognate peptides in the presence of SDS (Wucherpfennig 2003), an experimental system that may not be physiologically relevant. Other MHC haplotypes (DR2/DQ6) confer dominant protection against diabetes (Redondo et al. 2000). For instance, substituting proline for histidine at position 56 of I-A$^{g7}$ confers protection against diabetes (Wucherpfennig 2003). These data underscore the importance of MHC in antigen presentation in the development of type 1A diabetes.

*Antigen recognition*

Thymic selection of the T cell repertoire is key in many autoimmune diseases. Deletion or inactivation of self-reactive T cells depends on whether developing T cells "see" an autoantigen. Evidence for central tolerance failures in the development of diabetes is derived from various animal models and human autoimmune syndromes.

The polyendocrine disorder autoimmune-polyendocrinopathy-candidiasis-ectodermal dystrophy (APECED) or autoimmune polyendocrine syndrome type I (APS-I) are caused by mutations in the AIRE gene. The AIRE gene is expressed predominantly in the medullary epithelial cells of the thymus (with minor expression in peripheral lymphoid organs but none in parenchymal tissues) and influences ectopic expression of peripheral antigens (Anderson et al. 2002). Patients with APECED often have type 1 diabetes and produce insulin autoantibodies (Redondo et al. 2000). Mice lacking the *aire* protein do not express proinsulin in the thymus, show an increased number of activated memory T cells, and display an autoimmune profile similar to mice subjected to neonatal thymectomy (Anderson et al. 2002).

The thymus deletes autoreactive T cells in a dose-dependent manner (Anjos and Polychronakos 2004). Therefore, expression levels of proteins in the thymus can also influence whether a T cell is clonally deleted or allowed to mature. A variable number of tandem repeats (VNTR) of a 14-15 base pair sequence found upstream of the human insulin gene (INS) affects proinsulin messenger RNA levels in the thymus. Short alleles are associated with developing type 1 diabetes, and long alleles are associated with a lower risk of type1 diabetes (Matejkova-Behanova et al. 2004, Tait et al. 2004). Thymic expression of proinsulin is directly related to gene copy number (Chentoufi and Polychronakos 2002). The INS VNTR polymorphism also affects the levels of GAD65 autoantibodies in adults with new-onset diabetes and the polymorphism has a high positive predictive value for future insulin dependency (Zimmet et al. 1999). This VNTR is specific for autoimmune diabetes susceptibility only; it is not a marker for other autoimmune diseases like multiple sclerosis or Graves Disease (Tait et al. 2004).

As in the periphery, MHC molecules play a role in antigen presentation in the thymus. Diabetes in the NOD mouse has been prevented by expressing proinsulin 2 in MHC Class II cells (APCs) in the thymus and spleen (French et al. 1997).

*Antibody production*

The third immune cell required for type 1 diabetes in the NOD mouse is the B-lymphocyte. As stated above, the autoantibodies in human disease are considered to be markers of disease rather than primarily pathogenic. In the NOD mouse strains, antibodies are associated with insulitis, but the insulitis may or may not progress to diabetes (Robles et al. 2002). Greeley found that elimination of maternal autoantibodies prevented disease in genetically susceptible offspring (Greeley et al. 2002). Since B-lymphocytes serve multiple functions (antibody producers or antigen presenters), B-lymphocytes might be pathogenic in their role as APCs. In the NOD mouse, B-lymphocytes that present beta-cell antigens are critical for *in vivo* priming of islet antigens (Serreze et al. 1998, Noorchashm et al. 2003).

### Preproinsulin, proinsulin, and insulin

The initial mRNA transcript for insulin is for preproinsulin. After the signal sequence that directs the message to the endoplasmic reticulum is cleaved, proinsulin is packaged into secretory granules for export from the pancreas. Inside the secretory granule, proinsulin is cleaved to insulin and C-peptide, and the two molecules are present in a 1:1 ratio (Hutton et al.). Proinsulin is an early autoantigen in the development of diabetes in both mice and man (Ott et al. 2004).

Mice have two insulin genes (Ins1 and Ins2) on chromosomes 19 and 7, respectively, whereas humans possess only one insulin gene (INS) on chromosome 11 that is homologous to Ins2. The two proteins are very similar in structure; the mRNAs vary only by two amino acids in the B chain and several in the C peptide and leader sequences of preproinsulin (Thebault-Baumont et al. 2003, Moriyama et al. 2003). The Ins2 gene is expressed at much higher levels in the thymus compared to insulin 1 which is essentially absent from the thymus (Moriyama et al. 2003). Overall thymic expression of proinsulin is forty times lower than pancreatic insulin expression level (Chen et al. 2001). In young NOD mice, proinsulin message is expressed in the thymus at similar levels to non-autoimmune mice (Balb/c and B6), but only splenic T cells from young NOD mice can be stimulated by proinsulin peptide B24-C33 (Chen et al. 2001). This particular epitope is not found in insulin since the C-peptide is spliced from proinsulin. In older mice (6–8 weeks), the proliferative response to GAD65 equals that of proinsulin. GAD65 shares a 13 amino acid sequence homology with proinsulin BC peptide (Chen et al. 2001). Cross-reactivity between GAD65 and proinsulin peptides may be unlikely since a peptide library screen of GAD65 and proinsulin against T cells isolated from patients with type 1 diabetes and individuals with two or more autoantibodies failed to yield an immunodominant epitope of GAD65 consistent with cross-reactive priming between GAD65 and proinsulin (Ott et al. 2004).

Proinsulin 2 knockout mice (Ins2 −/−) develop diabetes, insulitis, and insulin autoantibodies at an accelerated rate compared to Ins2 +/+ mice and demonstrate an increased ability to transfer disease to naïve animals (Thebault-Baumont et al. 2003, Moriyama et al. 2003). Heterozygous knockout mice (Ins2 +/−) show accelerated disease over normal wild type NOD mice but less than homozygous knockout mice (Thebault-Baumont et al. 2003, Moriyama et al. 2003, Anjos and Polychronakos, 2004). In contrast, Ins1 knockout mice (Ins1 −/−) are protected from diabetes but not from sialitis or development of insulin autoantibodies (Moriyama et al. 2003). Ins1 may be a preferred target for peripheral anti-insulin autoimmunity whereas the presence or absence of thymic Ins2 may determine whether autoimmunity develops or not (Moriyama et al. 2003). Both native murine insulin sequences can be recognized by the immune system depending on specific histocompatibility alleles.

*What are the epitopes?*

Many putative epitopes of insulin and proinsulin have been identified in humans and mice using a variety of techniques. Peptides have been eluted from HLA molecules and sequenced via mass spectroscopy (Lieberman et al. 2003). Screening peptide libraries have been used to measure IFN gamma response of peripheral blood mononuclear cells (PBMCs) isolated from patients with diabetes (Ott et al. 2004). T cell clones have been isolated by using whole islets as antigens (Wegmann et al. 1994). *In vitro* stimulation of splenocytes with specific peptides have been used in proliferation assays (Chen et al. 2001) and mice have been challenged/immunized *in vivo* with specific peptides (Moriyama et al. 2003) or received transplants of islets expressing particular antigens (Faideau et al. 2004). T cell receptor transgenic mice models have been created (Gregersen et al. 2004).

It is still undetermined which epitope may be first or if there is a single consistent initiating epitope among all cases of diabetes. It is possible that the multiplicity of antigenic determinants from a single protein is the result of epitope spreading, i.e. a single epitope is the target of the precipitating event within the inflammatory milieu of the pancreas, not easily identified by examining peripheral blood lymphocytes (Ott et al. 2004), with epitope spreading occurring later in a second wave of autoimmunity. We hypothesize that the initial islet peptide recognized will be similar for individuals with the same MHC alleles, but the priming event will be one of a host of possible triggering events. Of note, immunization of NOD mice with the B:9-23 sequence from insulin 2 prevents diabetes whereas the B:9-23 peptide from insulin 1 does not (Devendra et al. 2004). The insulin 1 B:9-23 peptide differs from the insulin 2 peptide by a single amino acid (proline versus serine at position B9). When NOD mice expressing the B7.1 costimulatory molecule on islets are immunized with the insulin 1 peptide, diabetes is rapidly induced and when immunized with the insulin 2 peptide, these mice have a slower disease onset (Devendra et al. 2004). As might be expected, the response to the B:9:23 peptide is MHC-restricted (I-$A^{g7}$ and I-$A^{d}$ respond but I-$A^{b}$ does not).

Figure 2 shows several putative epitopes from preproinsulin in mouse models. Various experiments have identified the following CD4 epitopes in mice: B:2-16 (Halbout et al. 2002), A:7-21 (Daniel and Wegmann 1996), A:1-15 (Halbout et al. 2002), and B:9-23 (Abiru et al. 2000). CD8 T cells as well as CD4 T cells are required for development of T1DM (Lieberman et al. 2003), and CD8 T cells may be particularly important for the initiation of disease (Stene et al. 2004). CD8 T cell clones that bind B:15-22 and B:15-23 have been isolated and can transfer disease in mouse models (Wong et al. 1999). Most recently, NOD mice with both insulin genes knocked out and replaced with a single amino acid-substituted insulin 2 gene failed to develop autoimmune diabetes (Nakayama 2005).

In man, an HLA-DR-restricted T cell clone from a new-onset patient was found to react with B:11-27 (Schloot et al. 1998). This peptide includes the B:9-23 peptide that is so important in the MHC-restricted mouse model. The B:9-23 peptide was also found to stimulate T cell proliferation in recent-onset diabetic patients but not in age- or HLA-matched controls (Alleva et al. 2001). Single T cells cloned from pancreatic draining lymph nodes isolated from DR4 type 1 diabetic patients recognize the A: 1-15 insulin peptide (Kent 2005). Using enzyme-linked immunosorbent spot assays (ELISPOT), Peakman

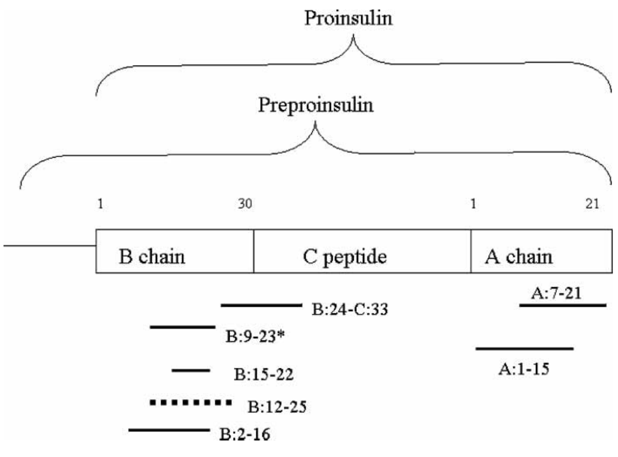

Figure 2. Linear structure of proinsulin message. The various putative murine epitopes of preproinsulin are shown as bars below the structure. The dashed line shows the epitope from a T cell clone that reacts with insulin but prevents diabetes. See text for references. *B:9-23 from both proinsulin1 and proinsulin2 prevent or provoke diabetes in the mouse.

and coworkers searched for and identified proinsulin-specific peptides to which diabetics and controls respond differentially. Diabetic patients respond with a pro-inflammatory phenotype whereas controls respond with a regulatory phenotype (Arif et al. 2004). Insulin, proinsulin, and several insulin peptides were found to stimulate B and T cells in type 1 diabetic patients and antibody-positive patients, both with DRB1*04 and DQB1*0302 haplotypes, further emphasizing the need to consider antigen- and MHC-specificity together (Durinovic-Bello et al. 2003).

*Prevention of disease by insulin*

Using insulin as a prophylactic treatment was tried because exogenous insulin might allow the pancreas to "rest," be able to induce peripheral tolerance, or ameliorate pancreatic toxicity of excess glucose (Herold 2004). Subcutaneous, intranasal, and intravenous insulin can either delay the onset or reduce the incidence of diabetes in animal models (Gotfredsen et al. 1985, Daniel and Wegmann 1996, Zekzer et al. 1997). Administration of the insulin peptide B:9-23 has been both provocative as well as preventive in mouse models (Moriyama et al. 2002, Liu et al. 2003, Stene et al. 2004) depending on the genetic background. Since the peptide does not have the physiological activity of insulin, tolerance induction might be the mechanism of disease prevention with B:9-23. In humans, the diabetes prevention trial (DPT-1) failed to find any effect of parenteral insulin, and the oral trial results have just been published with a subset showing potential efficacy (Skyler et al. 2005). Intranasal or inhaled trials of insulin are on-going. Pilot studies show better results in patients with normal FPIR (Daaboul and Schatz 2003).

### Conclusion

Type 1A diabetes is a complex disorder involving multiple genes and environmental influences. In human and animal disease, autoantibodies precede overt disease and may be used to identify individuals at risk of developing diabetes. The time between antibody appearance and onset of disease provides a therapeutic window of opportunity for preventing disease. The complexity of the disorder makes it likely that there are many alternative pathways that lead to autoimmune diabetes, but loss of central or peripheral tolerance to insulin is likely a key event in the pathogenesis of type 1A diabetes.

### Acknowledgements

This work was supported by grants from the National Institutes of Health (DK32083, AI39213, DK55969, DK62718, AI50864, AI95380, DK32493, DK50970, AI46374), Diabetes Endocrine Research Center (P30 DK57516), Clinical Research Centers (MO1 RR00069, MO1 RR00051), the American Diabetes Association, the Juvenile Diabetes Foundation, and the Children's Diabetes Foundation.

### References

Abiru N, Wegmann D, Kawasaki E, Gottlieb P, Simone E, Eisenbarth GS. 2000. Dual overlapping peptides recognized by insulin peptide B:9-23 reactive T cell receptor AV13S3 T cell clones of the NOD mouse. J Autoimmun 14:231–237.

Alleva DG, Crowe PD, Jin L, et al. 2001. A disease-associated cellular immune response in type 1 diabetics to an immunodominant epitope of insulin. J Clin Investig 107:173–180.

Anderson MS, Venanzi ES, Klein L, et al. 2002. Projection of an immunological self-shadow within the thymus by the aire protein. Science 298:1395–1401.

Anjos S, Polychronakos C. 2004. Mechanisms of genetic susceptibility to type I diabetes: Beyond HLA. Mol Genet Metab 81:187–195.

Arif S, Tree TI, Astill TP, et al. 2004. Autoreactive T cell responses show proinflammatory polarization in diabetes but a regulatory phenotype in health. J Clin Investig 113:451–463.

Chen W, Bergerot I, Elliott JF, et al. 2001. Evidence that a peptide spanning the B–C junction of proinsulin is an early autoantigen epitope in the pathogenesis of type 1 diabetes. J Immunol 167:4926–4935.

Chentoufi AA, Polychronakos C. 2002. Insulin expression levels in the thymus modulate insulin-specific autoreactive T-cell tolerance: The mechanism by which the IDDM2 locus may predispose to diabetes. Diabetes 51:1383–1390.

Daaboul J, Schatz D. 2003. Overview of prevention and intervention trials for type 1 diabetes. Rev Endocr Metab Disord 4:317–323.

Daniel D, Wegmann DR. 1996. Protection of nonobese diabetic mice from diabetes by intranasal or subcutaneous administration of insulin peptide B-(9-23). Proc Natl Acad Sci USA 93:956–960.

Devendra D, Paronen J, Moriyama H, Miao D, Eisenbarth GS, Liu E. 2004. Differential immune response to B:9-23 insulin 1 and insulin 2 peptides in animal models of type 1 diabetes. J Autoimmun 23:17–26.

Dotta F, Gianani R, Previti M, et al. 1996. Autoimmunity to the GM2-1 islet ganglioside before and at the onset of type I diabetes. Diabetes 45:1193–1196.

Durinovic-Bello I, Maisel N, Schlosser M, et al. 2003. Relationship between T and B cell responses to proinsulin in human type 1 diabetes. Ann NY Acad Sci 1005:288–294.

Eisenbarth GS, Nayak RC, Rabinowe SL. 1988. Type I diabetes as a chronic autoimmune disease. J Diabetes Complicat 2:54–58.

Eskola V, Vahasalo P, Akerblom HK, Knip M. 2003. Increased frequency of islet cell antibodies in unaffected brothers of children with type 1 diabetes. Horm Res 59:195–200.

Faideau B, Briand JP, Lotton C, et al. 2004. Expression of preproinsulin-2 gene shapes the immune response to preproinsulin in normal mice. J Immunol 172:25–33.

French MB, Allison J, Cram DS, et al. 1997. Transgenic expression of mouse proinsulin II prevents diabetes in nonobese diabetic mice. Diabetes 46:34–39.

Gotfredsen CF, Buschard K, Frandsen EK. 1985. Reduction of diabetes incidence of BB Wistar rats by early prophylactic insulin treatment of diabetes-prone animals. Diabetologia 28:933–935.

Graves PM, Rotbart HA, Nix WA, et al. 2003. Prospective study of enteroviral infections and development of beta-cell autoimmunity. Diabetes Autoimmunity Study in the Young (DAISY). Diabetes Res Clin Pract 59:51–61.

Greeley SA, Katsumata M, Yu L, et al. 2002. Elimination of maternally transmitted autoantibodies prevents diabetes in nonobese diabetic mice. Nat Med 8:399–402.

Gregersen JW, Holmes S, Fugger L. 2004. Humanized animal models for autoimmune diseases. Tissue Antigens 63:383–394.

Halbout P, Briand JP, Becourt C, Muller S, Boitard C. 2002. T cell response to preproinsulin I and II in the nonobese diabetic mouse. J Immunol 169:2436–2443.

Herold KC. 2004. Treatment of type 1 diabetes mellitus to preserve insulin secretion. Endocrinol Metab Clin North Am 33:93.

von Herrath M, Bach JF. 2002. Juvenile autoimmune diabetes: A pathogenic role for maternal antibodies? Nat Med 8:331–333.

Hummel M, Bonifacio E, Schmid S, Walter M, Knopff A, Ziegler AG. 2004. Brief communication: Early appearance of islet autoantibodies predicts childhood type 1 diabetes in offspring of diabetic parents. Ann Intern Med 140:882–886.

Hutton JC, Eisenbarth GS. 2003. A pancreatic {beta}-cell-specific homolog of glucose-6-phosphatase emerges as a major target of cell-mediated autoimmunity in diabetes. Proc Natl Acad Sci USA 100:8626–8628.

Hutton J, Wasmeier C, Amaria R, Bright N, Creemers J, Proprotein processing and pancreatic islet function. Type 1 Diabetes: Molecular, Cellular and Clinical Immunology

Juhl K, Hutton J, Stimulus-secretion coupling in the pancreatic beta-cell. Type 1 Diabetes: Molecular, Cellular and Clinical Immunology. http://www.uchsc.edu/misc/diabetes/books.html

Kent S, Chen Y, Breglio L, et al. 2005. Expanded T cells from pancreatic lymph nodes of type 1 diabetic subjects recognize an insulin epitope. Nat 435:224–228.

Lieberman SM, Evans AM, Han B, et al. 2003. Identification of the {beta} cell antigen targeted by a prevalent population of pathogenic CD8+ T cells in autoimmune diabetes. Proc Natl Acad Sci USA 100:8384–8388.

Liu E, Moriyama H, Paronen J, et al. 2003. Nondepleting anti-CD4 monoclonal antibody prevents diabetes and blocks induction of insulin autoantibodies following insulin peptide B:9-23 immunization in the NOD mouse. J Autoimmun 21:213–219.

Marner B, Lernmark A, Ludvigsson J, et al. 1985. Islet cell antibodies in insulin-dependent (type 1) diabetic children treated with plasmapheresis. Diabetes Res 2:231–236.

Martin S, Wolf-Eichbaum D, Duinkerken G, et al. 2001. Development of type 1 diabetes despite severe hereditary B-lymphocyte deficiency. N Engl J Med 345:1036–1040.

Matejkova-Behanova M, Vankova M, Hill M, et al. 2004. Polymorphism of INS VNTR is associated with glutamic acid decarboxylase antibodies and postprandial C-peptide in patients with onset of diabetes after 35 years of age. Physiol Res 53:187–190.

Milgrom F, Witebsky E. 1962. Autoantibodies and autoimmune disease. JAMA 181:706–716.

Moriyama H, Wen L, Abiru N, et al. 2002. Induction and acceleration of insulitis/diabetes in mice with a viral mimic (polyinosinic-polycytidylic acid) and an insulin self-peptide. Proc Natl Acad Sci USA 99:5539–5544.

Moriyama H, Abiru N, Paronen J, et al. 2003. Evidence for a primary islet autoantigen (preproinsulin 1) for insulitis and diabetes in the NOD mouse. Proc Natl Acad Sci USA 100:10376–10381.

Nakayama M, Abiru N, Wegmann D, et al. 2005. Prime role for an insulin epitope in the development of type 1 diabetes in NOD mice. Nat 435:220–223.

Noorchashm H, Greeley SA, Naji A. 2003. The role of T/B lymphocyte collaboration in the regulation of autoimmune and alloimmune responses. Immunol Res 27:443–450.

Norris JM, Barriga K, Klingensmith G, et al. 2003. Timing of cereal exposure in infancy and risk of islet autoimmunity. The Diabetes Autoimmunity Study in the Young (DAISY). JAMA 290:1713–1720.

Ott PA, Dittrich MT, Herzog BA, et al. 2004. T cells recognize multiple GAD65 and proinsulin epitopes in human type 1 diabetes, suggesting determinant spreading. J Clin Immunol 24:327–339.

Redondo MJ, Kawasaki E, Mulgrew CL, et al. 2000. DR and DQ associated protection from type 1 diabetes: Comparison of DRB1*1401 and DQA1*0102-DQB1*0602. J Clin Endocrinol Metab 85:3793–3797.

Robles DT, Eisenbarth GS, Dailey NJM, Peterson LB, Wicker LS. 2002. Insulin autoantibodies are associated with islet inflammation but not always related to diabetes progression in NOD congenic mice. Diabetes 52:882–886.

Sairenji T, Daibata M, Sorli CH, et al. 1991. Relating homology between the Epstein-Barr virus BOLF1 and HLA-DQw8 beta chain to recent onset type 1 (insulin-dependent) diabetes mellitus. Diabetologia 34:33–39.

Schloot NC, Willemen S, Duinkerken G, De Vries RRP, Roep BO. 1998. Cloned T cells from a recent onset IDDM patient reactive with insulin B-chain. J Autoimmun 11:169–175.

Serreze DV, Fleming SA, Chapman HD, Richard SD, Leiter EH, Tisch RM. 1998. B lymphocytes are critical antigen-presenting cells for the initiation of T cell-mediated autoimmune diabetes in nonobese diabetic mice. J Immunol 161:3912–3918.

Skyler J, Krischer J, Wolfsdorf J, Coure C, Palmer J, Greenbaum C, Cathbertson D, Rafkin-Mervis E, Chase H, Leschek, E. 2005. Effects of oral insulin in relatives of patients with type 1 diabetes: The Diabetes Prevention Trial—Type 1. Diabetes Care May 28(5): 1068–1076.

Stene LC, Barriga K, Norris JM, et al. 2004. Perinatal factors and development of islet autoimmunity in early childhood: The diabetes autoimmunity study in the young. Am J Epidemiol 160:3–10.

Tait KF, Collins JE, Heward JM, et al. 2004. Evidence for a Type 1 diabetes-specific mechanism for the insulin gene-associated IDDM2 locus rather than a general influence on autoimmunity. Diabet Med 21:267–270.

Thebault-Baumont K, Dubois-LaForgue D, Krief P, et al. 2003. Acceleration of type 1 diabetes mellitus in proinsulin 2-deficient NOD mice. J Clin Invest 111:851–857.

Verge CF, Gianani R, Kawasaki E, et al. 1996. Prediction of type I diabetes in first-degree relatives using a combination of insulin, GAD, and ICA512bdc/IA-2 autoantibodies. Diabetes 45:926–933.

Verge CF, Stenger D, Bonifacio E, et al. 1998. Combined use of autoantibodies (IA-2 autoantibody, GAD autoantibody, insulin autoantibody, cytoplasmic islet cell antibodies) in type 1 diabetes: Combinatorial islet autoantibody workshop. Diabetes 47:1857–1866.

Wegmann DR, Norbury-Glaser M, Daniel D. 1994. Insulin-specific T-cells are a predominant component of islet infiltrates in prediabetic NOD mice. Eur J Immunol 24:1853–1857.

Wong FS, Karttunen J, Dumont C, et al. 1999. Identification of an MHC class I-restricted autoantigen in type 1 diabetes by screening an organ-specific cDNA library. Nat Med 5:1026–1031.

Wucherpfennig KW. 2003. MHC-Linked susceptibility to type 1 diabetes a structural perspective, p 119–127.

Yu L, Eisenbarth GS, Humoral Autoimmunity in Type 1 Diabetes: Cellular, Molecular and Clinical Immunology. Type 1 Diabetes: Molecular, Cellular and Clinical Immunology http://www.uchsc.edu/misc/diabetes/books.html

Yu L, Rewers M, Gianani R, et al. 1996. Anti-islet autoantibodies develop sequentially rather than simultaneously. J Clin Endocrinol Metab 81:4264–4267.

Zekzer D, Wong FS, Wen L, et al. 1997. Inhibition of diabetes by an insulin-reactive CD4 T-cell clone in the nonobese diabetic mouse. Diabetes 46:1124–1132.

Zimmet P, Turner R, McCarty D, Rowley M, Mackay I. 1999. Crucial points at diagnosis—Type 2 diabetes or slow type 1 diabetes. Diabetes Care 22:B59–B64.

